# Muscle Fatigue Analysis With Optimized Complementary Ensemble Empirical Mode Decomposition and Multi-Scale Envelope Spectral Entropy

**DOI:** 10.3389/fnbot.2020.566172

**Published:** 2020-11-05

**Authors:** Juan Zhao, Jinhua She, Edwardo F. Fukushima, Dianhong Wang, Min Wu, Katherine Pan

**Affiliations:** ^1^School of Automation, China University of Geosciences, Wuhan, China; ^2^Hubei Key Laboratory of Advanced Control and Intelligent Automation for Complex Systems, Wuhan, China; ^3^School of Engineering, Tokyo University of Technology, Tokyo, Japan; ^4^Division of Biology and Biological Engineering, California Institute of Technology, Pasadena, CA, United States

**Keywords:** surface electromyography, complementary ensemble empirical mode decomposition, least-squares mutual information, multi-scale envelope spectral entropy, muscle fatigue

## Abstract

The preprocessing of surface electromyography (sEMG) signals with complementary ensemble empirical mode decomposition (CEEMD) improves frequency identification precision and temporal resolution, and lays a good foundation for feature extraction. However, a mode-mixing problem often occurs when the CEEMD decomposes an sEMG signal that exhibits intermittency and contains components with a near-by spectrum into intrinsic mode functions (IMFs). This paper presents a method called optimized CEEMD (OCEEMD) to solve this problem. The method integrates the least-squares mutual information (LSMI) and the chaotic quantum particle swarm optimization (CQPSO) algorithm in signal decomposition. It uses the LSMI to calculate the correlation between IMFs so as to reduce mode mixing and uses the CQPSO to optimize the standard deviation of Gaussian white noise so as to improve iteration efficiency. Then, useful IMFs are selected and added to reconstruct a de-noised signal. Finally, considering that the IMFs contain abundant frequency and envelope information, this paper extracts the multi-scale envelope spectral entropy (MSESEn) from the reconstructed sEMG signal. Some original sEMG signals, which were collected from experiments, were used to validate the methods. Compared with the CEEMD and complete ensemble empirical mode decomposition with adaptive noise (CEEMDAN), the OCEEMD effectively suppresses mode mixing between IMFs with rapid iteration. Compared with approximate entropy (ApEn) and sample entropy (SampEn), the MSESEn clearly shows a declining tendency with time and is sensitive to muscle fatigue. This suggests a potential use of this approach for sEMG signal preprocessing and the analysis of muscle fatigue.

## 1. Introduction

Muscle fatigue is defined as a temporary decrease in the physical force during exercise (Liu et al., 2014; Kyranou et al., 2018). During a continuous contraction, a localized muscle gradually undergoes biological changes and enters the state of muscle fatigue. While the mechanism of muscle fatigue is complicated, accurate detection of fatigue is of great significance for assessing functional impairment, planning training programs, and evaluating rehabilitation effect (Gandevia, 2013). For these reasons, the detection of muscle fatigue has been a hot topic in the field of rehabilitation and sports medicine over the last couple of decades.

A surface electromyography (sEMG) signal captures the state of muscle activity and motor function and is considered as an effective tool to evaluate local muscle fatigue (Chowdhury et al., 2013). The changes in sEMG signals correlate to the number of motor units, activity patterns, metabolic situations, and other factors (Srhoj-Egekher et al., 2011). An sEMG signal is non-stationary during muscle dynamic contractions and shows a high degree of complexity (Zhang Q. et al., 2017). Thus, it is a reliable approach to processing sEMG signals by a non-linear method and to extract muscle fatigue features from the complexity.

An essential part of processing a non-stationary signal is to find a way to represent the oscillatory modes of the signal. An sEMG signal consists of many single harmonic signals, and each contains only one oscillatory mode. Introduced by Huang et al., an intrinsic mode function (IMF) is a single harmonic signal model that gives sharp identifications of embedded structures through producing instantaneous frequencies as functions of time (Huang, 2000). With the empirical mode decomposition (EMD), a non-stationary signal is decomposed into IMFs and performed time-frequency analysis (Huang, 2000). The EMD was first used to filter the activity attenuation of sEMG signals (Andrade et al., 2006). Later, it was widely used for the artifact removal and feature extraction of sEMG signals (Pilkar et al., 2017). Unfortunately, the mode-mixing problem often occurs for the reason that the IMFs contain signals of different scales or the signals of a similar scale spread in different IMFs (Hu et al., 2012). To solve this problem, a method of analyzing noise-assisted signals called ensemble EMD (EEMD) was presented (Wu and Huang, 2009). The EEMD has been used not only to filter various noises (Zhang and Zhou, 2013) but also to quantitatively analyze the features of sEMG signals (Wu et al., 2017; Zhang Y. et al., 2017). This method has a good effect on suppressing mode mixing decomposed from weak discontinuous signals with high-frequency noise, but the suppression effect is very limited for signals with similar frequencies. Therefore, the EEMD may leave a small amount of noise in reconstructed signals.

Many methods have been proposed to further reduce noise for the EEMD, such as a complementary EEMD (CEEMD) (Yeh et al., 2010) and a complete EEMD with adaptive noise (CEEMDAN) (Torres et al., 2011). They improved the EEMD in different ways. The CEEMDAN adds adaptive white noise to the original signals and obtains IMFs by averaging the modal components at each stage of signal decomposition. However, the CEEMDAN still has some problems like spurious modes and high computational cost (Rezaie-Balf et al., 2019; Li et al., 2020). The CEEMD adds a pair of Gaussian white noises with equal amplitudes and a relative phase difference of 180° to the original signal, and then performs the EMD decomposition of the two groups of signals.

The CEEMD decomposes a signal into IMFs based on the characteristics of the signal itself, which is very important for analyzing a non-stationary signal. It was used in some fields and its applications demonstrate successful results (Zhao et al., 2014; Lu et al., 2019). Based on the advantages of the CEEMD, this paper employs it to preprocess sEMG signals, which lays a good foundation for next signal reconstruction, de-noising, and feature extraction. However, the CEEMD also has a small number of mode-mixing components and long iterative times when it decomposes a signal that exhibits intermittency and contains components with proximity spectrum into intrinsic mode functions (IMFs) (Zheng et al., 2014; Chen and Wang, 2017).

This paper analyzes the causes of these problems. On one hand, there is information coupling between IMFs, which leads to the existence of similar signal components in different IMFs. On the other hand, the standard deviation of added Gaussian white noise is not suitable. As a result, the signal decomposition is not complete, or the number of iterations increases in order to achieve a specific decomposition effect. Therefore, this paper presents an improved CEEMD to overcome these defects.

After the sEMG signal is preprocessed, the feature of evaluating muscle fatigue needs to be extracted. The quality of the feature has a crucial influence on the classification and predicting of muscle fatigue. The complexity of sEMG is a commonly used index to reflect the physiological characteristics of muscle activity (Talebinejad et al., 2011; Cashaback et al., 2013). Extracting entropy from sEMG is an effective method for analyzing muscle fatigue. Approximate entropy (ApEn) and sample entropy (SampEn) are the earliest entropy parameters and are still in use today (Pethick et al., 2019; Xie et al., 2019). However, they are subject to the problem of tolerance selection. Furthermore, Costa et al. found that the single-scale entropy of a healthy person often conflicts with that of a heart-disease patient for the use of their heartbeat fluctuations. To solve these problems, they proposed the concept of multi-scale entropy (MSEn), which is calculated on multiple time scales of a signal (Gao et al., 2015). Different time scales are obtained through a coarse granulation process, and provide more complete information on signals than a single scale does. Note that the evolution of muscle fatigue needs to be described in this study, the selected entropy-based indicator should be related to the characteristics of sEMG signals.

The purpose of this study was to devise a method, which is called optimized CEEMD (OCEEMD), to process sEMG signals with high precision and high efficiency, and to present a new entropy-based index for muscle fatigue. The OCEEMD integrates the least-squares mutual information (LSMI) (Kimura and Sugiyama, 2011) and the chaotic quantum particle swarm optimization (CQPSO) algorithm (Valdez et al., 2017) with the conventional CEEMD. The LSMI is used to calculate the correlation between IMFs to reduce their coupling. And the CQPSO is used to search an optimal solution of the standard deviation of Gaussian white noise to optimize a related parameter. The advantage of the OCEEMD is that it suppresses mode mixing and improves decomposition efficiency by making the best use of the algorithms. After the OCEEMD decomposes an sEMG signal into a set of IMFs adaptively, the useful IMFs are reconstructed to a de-noised signal, eliminating most of the interference. Then, multi-scale envelope spectral entropy (MSESEn) is calculated. This index improves the reliability of analyzing muscle fatigue based on the rich frequency and envelope information of IMFs. Verification was carried out for collected sEMG signals in a pedaling experiment for the presented and the related methods. The results show that, compared with other methods, the OCEEMD suppresses the mode mixing with high efficiency for the decomposition of sEMG signals, and the extracted feature shows a high sensitivity to the sEMG changes with muscle fatigue.

## 2. Materials and Methods

This section first introduces the experimental protocol and tests. Then, it describes signal preprocessing and feature extraction of an sEMG signal.

### 2.1. Subjects and sEMG Data Acquisition

Ten healthy subjects (two females and eight males) performed the experiments at the Advanced Mechatronics Laboratory, School of Engineering, Tokyo University of Technology, Japan (Table 1). They were covered by personal accident insurance for students pursuing education and research provided by Japan Educational Exchanges and Services. The advisability of students' involvement in experiments and the experimental protocol were first assessed by the ethical committee of Tokyo University of Technology. All participants signed informed consent.

**Table 1:** Information of 10 subjects involved in experiments (Min., Minimum; Max., Maximum; Avg., Average; Std. Dev., Standard Deviation).

	**Min**.	**Max**.	**Avg**.	**Std. Dev**.
Age (years)	23	39	29.7	12.1
Height (cm)	155.6	182.9	170.3	7.8
Weight (kg)	44	86.4	68.5	2.7

**Figure 1:**
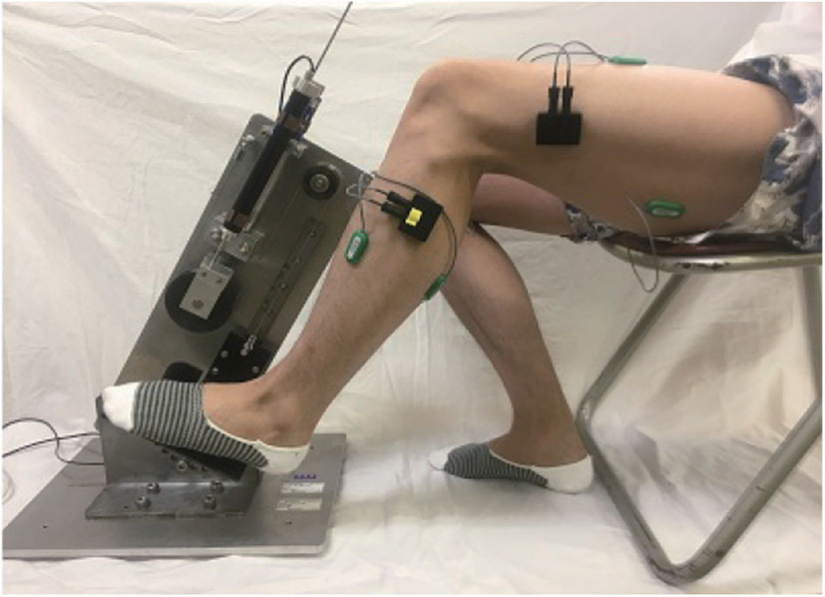
A shot of pedaling experiment.

Each subject sat in front of a pedaling machine that was developed for the rehabilitation training of lower limbs (Figure 1). The training load, inclined pedal angle, seat height, distance from the machine, and other parameters were calibrated to suit the subject before running the experiments. Each subject placed his/her left foot on the pedal for 5 min of up-down pedaling, with appropriate training load so as to feel local muscle fatigue after training. The sEMG data were collected from four muscles of lower limbs: rectus femoris, biceps femoris, tibialis anterior, and gastrocnemius (She et al., 2017a,b). Four electrodes (Model: Biometrics SX230-1000; Origin: UK) were used to collect sEMG signals, which were attached on the muscle belly along the muscle fibers. The sEMG signals were sampled at 1,000 Hz with a fixed gain of 60 dB (amplification factor: 1000). The data were stored in a laptop computer [Model: DELL Precision M3800; OS: Windows 8.1 Pro 64 bits; CPU: Intel(R) Core(TM) i7-4712HQ; RAM: 16.0 GB] (Zhao et al., 2018).

### 2.2. Optimized Complementary Ensemble Empirical Mode Decomposition

An original sEMG signal contains noise and invalid frequency components. So, we need to perform signal preprocessing to extract true information. An improved CEEMD called optimized CEEMD (OCEEMD) is used to decompose the original sEMG signal into a set of IMFs, solving the mode-mixing problem with high iteration efficiency. Then according to their frequency characteristics, the useful IMFs are selected to reconstruct a new signal, achieving the effect of de-noising.

The CEEMD decomposition has three main processes. First, a pair of Gaussian white noises that have the same amplitudes and a relative phase difference of 180° are added to an sEMG signal to form two new signals by making use of the uniform distribution property of the white-noise spectrum. Then, the two new signals are decomposed and their IMF candidates are obtained, separately. Finally, every two IMF candidates at the same level are averaged as the IMF component of the original signal, and thus the corresponding residual function is calculated (Yeh et al., 2010).

Since two similar white-noise signals are added to the sEMG signal with opposite phases, the residual noises in the IMFs are only different in their signs. They cancel with each other when the IMFs are added to form a new IMF that contains little noise. This method effectively suppresses mode mixing. However, it is difficult to completely eliminate mode mixing in actual applications. It is necessary to investigate the causes of mode mixing so that we can further reduce it.

#### 2.2.1. Least-Squares Mutual Information

Mode mixing occurs when the components of similar scales reside in different IMFs. At this time, there is information coupling between the IMFs, that is, the IMFs are not completely orthogonal to each other. So, the existence of information coupling between the IMFs is a primary cause of mode mixing.

Theoretically speaking, eliminating the coupling between IMF components ensures the perfect orthogonality of the IMF components. Thus, it solves the mode-mixing problem completely. Mutual information (MI) is a non-parametric and non-linear measure indicator that quantitatively represents the statistical correlation between two random variables in information theory (Valdez et al., 2017). Since an sEMG signal has a zero-mean characteristic, the MI can be used to measure the coupling degree of IMF components obtained by the CEEMD according to the equivalence principle of irrelevance and orthogonality of zero-mean random signals. That is, it is possible to use the MI to measure whether or not there is mode mixing and the level of mixing. Thus, the first improvement of the OCEEMD on the CEEMD is the embedding of the least-squares mutual information (LSMI) in the CEEMD.

For two given IMFs, ${\left\{x_{i}\right\}}_{i=1}^{n}$ and ${\left\{y_{j}\right\}}_{j=1}^{n}$, their LSMI is calculated as follows.

Let the marginal probabilities of *x*_*i*_ be and *y*_*j*_ be *p*(*x*_*i*_) and *p*(*y*_*j*_), respectively; and the joint probability of *x*_*i*_ and *y*_*j*_ be *p*(*x*_*i*_, *y*_*j*_). The MI of two IMFs is defined to be

(1) $$\begin{array}{l} \text{MI}=\overset{n}{\underset{i=1}{\sum }}\overset{n}{\underset{j=1}{\sum }}p\left(x_{i},y_{j}\right)\ln\;\psi \left(x_{i},y_{j}\right), \end{array}$$

where *ψ*(*x*_*i*_, *y*_*j*_) is a density-ratio function

(2) $$\begin{array}{l} \psi \left(x_{i},y_{j}\right)=\frac{p\left(x_{i},y_{j}\right)}{p\left(x_{i}\right)p\left(y_{j}\right)}. \end{array}$$

MI is non-negative. It is zero if *x_i_* and *y*_*j*_ are statistically uncorrelated, that is,

(3) $$\begin{array}{l} \psi \left(x_{i},y_{j}\right)=1. \end{array}$$

The logarithmic function in (1) is sensitive to a change in *ψ*(*x*_*i*_, *y*_*j*_) around one. This degrades the accuracy of MI around zero. Note that based on the Taylor's series approximation, when *ψ*(*x*_*i*_, *y*_*j*_) − 1 → 0,

(4) $$\begin{array}{l} \ln\;\left\{1+\left[\psi \left(x_{i},y_{j}\right)-1\right]\right\}\approx \psi \left(x_{i},y_{j}\right)-1 \end{array}$$

and

(5) $$\begin{array}{l} {\left\{1+\left[\psi \left(x_{i},y_{j}\right)-1\right]\right\}}^{2}\approx 1+2\left[\psi \left(x_{i},y_{j}\right)-1\right] \end{array}$$

hold. Combining (4) and (5) yields

(6) $$\begin{array}{l} \ln\;\psi \left(x_{i},y_{j}\right)\approx \frac{1}{2}\left[\psi^{2}\left(x_{i},y_{j}\right)-1\right] \end{array}$$

Thus,

(7) $$\begin{array}{l} p\left(x_{i},y_{j}\right)\ln\;\psi \left(x_{i},y_{j}\right)\approx \frac{1}{2}p\left(x_{i}\right)p\left(y_{j}\right){\left[\psi \left(x_{i},y_{j}\right)-1\right]}^{2} \end{array}$$

when *ψ*(*x*_*i*_, *y*_*j*_) → 1.

Substituting (7) into (1) gives a squared-loss mutual information (SMI) (Kimura and Sugiyama, 2011)

(8) $$\begin{array}{l} \text{SMI}=\frac{1}{2}\overset{n}{\underset{i=1}{\sum }}\overset{n}{\underset{j=1}{\sum }}{\left[\psi \left(x_{i},y_{j}\right)-1\right]}^{2}p\left(x_{i}\right)p\left(y_{j}\right). \end{array}$$

An equivalent form of (8) is used to simplify the calculation:

(9) $$\begin{array}{l} \frac{1}{2}\overset{n}{\underset{i=1}{\sum }}\overset{n}{\underset{j=1}{\sum }}p\left(x_{i},y_{j}\right)\psi \left(x_{i},y_{j}\right)-\frac{1}{2}. \end{array}$$

Since neither *p*(*x*_*i*_), *p*(*y*_*j*_), nor *p*(*x*_*i*_, *y*_*j*_) is known, LSMI is used to further approximately calculate *ψ*(*x*_*i*_, *y*_*j*_) so as to approximate SMI from a paired data set of *x*_*i*_ and *y*_*j*_: (*x*_1_, *y*_1_), ⋯ , (*x*_*n*_, *y*_*n*_) (Kimura and Sugiyama, 2011). In this study, *ψ*(*x*_*i*_, *y*_*j*_) was approximated by a Gaussian-radial-basis-function model

(10) $$\begin{array}{l} \psi_{\Theta }=\overset{n}{\underset{i=1}{\sum }}\Theta_{i}K\left(x_{i},y_{j}\right), \end{array}$$

where Θ_*i*_ (*i* = 1, …, *n*) are estimated parameters and

(11) $$\begin{array}{l} K\left(x_{i},y_{j}\right)=\exp\left(-\frac{∥x_{i}-y_{i}{∥}_{2}^{2}}{2h^{2}}\right) \end{array}$$

is a kernel function. *h* in (11) is the width of the kernel.

The problem of optimizing the squared error of the estimated parameters using an empirical approximation is defined to be

(12) $$\begin{array}{l} \underset{\Theta }{\min}\left\{\frac{1}{2}\Theta^{T}\hat{H}\Theta -\Theta^{T}\hat{h}+\frac{\lambda }{2}∥\Theta {∥}^{2}\right\}, \end{array}$$

where λ is a regularization parameter; and *Ĥ* and *ĥ* are an *n* × *n* matrix and an *n*-dimensional vector, respectively. They are

(13) $$\begin{array}{l} \{\begin{array}{l} \hat{H}=\frac{1}{n^{2}}\overset{n}{\underset{i=1}{\sum }}\overset{n}{\underset{j=1}{\sum }}K\left(x_{i},y_{j}\right)K{\left(x_{i},y_{j}\right)}^{T},\\ \hat{h}=\frac{1}{n}\overset{n}{\underset{i=1}{\sum }}\overset{n}{\underset{j=1}{\sum }}K\left(x_{i},y_{j}\right). \end{array} \end{array}$$

The analytical solution of (12) is

(14) $$\begin{array}{l} \hat{\Theta }={\left(\hat{H}+\lambda I\right)}^{-1}\hat{h}, \end{array}$$

where *I* is the identity matrix. Thus, LSMI is given by

(15) $$\begin{array}{l} \text{LSMI}=\frac{1}{2}{\hat{h}}^{T}{\left(\hat{H}+\lambda I\right)}^{-1}\hat{h}-\frac{1}{2}, \end{array}$$

which is used to approximate SMI. Note that LSMI between two IMFs can be calculated directly. If all LSMIs for an sEMG signal are sufficiently small, all of the IMFs are orthogonal to each other. This means that there is no mode mixing.

#### 2.2.2. Chaotic Quantum Particle Swarm Optimization

The standard deviation of the added Gaussian white noise is an important parameter in the CEEMD. An unsuitable value may result in a large number of iterations and IMF modes and may cause mode mixing. So, the unsuitable standard deviation of Gaussian white noise is another cause of mode mixing.

Let *d* be the standard deviation of the added white noise. The selection of *d* requires careful consideration. As a rule of thumb, *d* is usually chosen to be a value between 0.1 and 0.2 (Torres et al., 2011). But choosing a different number between 0.1 and 0.2 inevitably leads to different decomposition results. The chaotic quantum particle swarm optimization (CQPSO) is an evolutionary computational algorithm (Huang, 2016). The main purpose of the algorithm is to obtain an optimal solution by sharing information among individuals of particle populations. It can be used here to search for the optimal solution of *d*. Thus, the second improvement of the OCEEMD on CEEMD is the embedding of CQPSO in the CEEMD to search for an optimal *d*.

LSMI is used as a fitness function for the CQPSO:

(16) $$\begin{array}{l} \arg\;\underset{d}{\min}\text{LSMI}. \end{array}$$

A minimum LSMI corresponds to an optimal *d*, which is used to determine the amplitudes of the white noise.

At the beginning of the *t*th iteration for a *M*-dimensional space with *N* individuals, let the location of the *i*th particle be $X_{i}={\left\{X_{ij}\right\}}_{j=1}^{M}\;\left(i=1,2,\ldots ,N\right)$, the historical location of the *i*th particle be $P_{i}={\left\{P_{ij}\right\}}_{j=1}^{M}$, and the searched optimal locations of particle populations be $P_{k}^{\left(t\right)}={\left\{P_{kj}^{\left(t\right)}\right\}}_{j=1}^{M}\;\left(k=1,2,\ldots ,N\right)$. The CQPSO of searching for an optimal *d* has the following steps.

Step 1: Initialize the particle population by chaos and randomly generate a series of parameters for *d* with *N* individuals. Initialize the locations of the particles.
Step 2: On the *t*th iteration, calculate the fitness of each particle, LSMI, at different locations, and compare each with the corresponding historical optimal fitness. If the current fitness is smaller than the historical one, replace the location vector *P*_*i*_ by the current one, *X*_*i*_. Otherwise, keep *P*_*i*_ unchanged.
Step 3: Determine the optimal locations $P_{k}^{\left(t\right)}$ by comparing all particles with their corresponding optimal fitness—the minimum of LSMI.
Step 4: Update the location of each particle and calculate the corresponding fitness like Step 2. Retain the particle with the best performance in the population according to the fitness function. Then, update *P*_*i*_ and $P_{k}^{\left(t\right)}$.
Step 5: Check whether or not the current *d* meets the preset accuracy requirement and if the number of iteration is larger than a prescribed one. If not, let *t* = *t* + 1 and go to Step 2. Otherwise, stop and output the solution of *d* that corresponds to the optimal location $P_{k}^{\left(t\right)}$.

The OCEEMD incorporating the LSMI and CQPSO reduces mode mixing and improves iteration efficiency. It is used to decompose an sEMG signal to IMFs with real physical significance and a final residual according to a time scale. Then, all useful IMFs are selected and added to reconstruct a new signal in which most interference is removed.

### 2.3. Multi-Scale Envelope Spectral Entropy

A multi-scale entropy is a sample entropy measurement on multiple time scales and can analyze the complexity of a signal on different scales (Costa et al., 2008). Consequently, it provides complete information about an sEMG signal. The envelope spectrum is a spectral analysis method that is sensitive to shock components of a signal and reflects sudden changes in an sEMG signal (Lv et al., 2015). Since IMF components after the OCEEMD decomposition preserve rich information about frequency and envelope, we use multi-scale envelope spectral entropy (MSESEn) as an index for the detection of muscle fatigue.

The calculation of the MSESEn has two main steps: First, calculate the envelope spectrum of a signal. Second, calculate the multi-scale entropy (MSEn) of the envelope spectrum. The steps are explained as follows:

In the first step, we carry out the Hilbert-Huang transformation (HHT) on a reconstructed signal, *f*(*t*),

(17) $$\begin{array}{l} H\left(f\left(t\right)\right)=\frac{1}{\pi }\int_{-\infty }^{+\infty }\frac{f\left(s\right)}{t-s}ds. \end{array}$$

Structuring an analytic function yields the envelope of the signal

(18) $$\begin{array}{l} B\left(t\right)=\sqrt{f\left(t\right)+H^{2}\left(f\left(t\right)\right)}. \end{array}$$

Then, we use the fast Fourier transform (FFT) to obtain the demodulation spectrum

(19) $$\begin{array}{l} B\left(\omega \right)=\text{FFT}\left(B\left(t\right)\right). \end{array}$$

In the second step, dividing a sequence of an envelope spectrum that contains *N* data points, ${\left\{z_{i}\right\}}_{i=1}^{N}$, into *K* segments with a scale τ, that is, ${\left\{z_{j},z_{j+1},\ldots ,z_{j+\tau }\right\}}_{j=1}^{K}$, where *K* is the integer part of *N*/τ. The series of continuous coarse granulation is ${\left\{w_{j}^{\left(\tau \right)}\right\}}_{j=1}^{K}$ and

(20) $$\begin{array}{l} w_{j}^{\left(\tau \right)}=\frac{1}{\tau }\overset{j\tau }{\underset{i=\left(j-1\right)\tau +1}{\sum }}z_{j},\;1\leq j\leq K. \end{array}$$

Then, for the sequence ${\left\{w_{j}^{\left(\tau \right)}\right\}}_{j=1}^{K}$, embedding *m* adjacent points constructs a new sequence, ${\left\{w_{k}^{\left(\tau \right)},w_{k+1}^{\left(\tau \right)},\ldots ,w_{k+m}^{\left(\tau \right)}\right\}}_{k=1}^{K-m+1}$. The distance $d_{i,j}^{\left(\tau \right)}$ between different elements, $\left(w_{i}^{\left(\tau \right)},w_{i+1}^{\left(\tau \right)},\ldots ,w_{i+m}^{\left(\tau \right)}\right)$ and $\left(w_{j}^{\left(\tau \right)},w_{j+1}^{\left(\tau \right)},\ldots ,w_{j+m}^{\left(\tau \right)}\right)$, is calculated for all *i* ≠ *j*. For a threshold γ, let the number of the distances satisfying $d_{i,j}^{\left(\tau \right)}\leq \gamma$ be *n*^(τ, *m*)^. The ratio of *n*^(τ, *m*)^ to *K* − *m* + 1 is

(21) $$\begin{array}{l} C_{i}^{\left(\tau ,m\right)}\left(\gamma \right)=\frac{n^{\left(\tau ,m\right)}}{K-m+1}. \end{array}$$

The correlation degree between different vector elements is

(22) $$\begin{array}{l} C^{\left(\tau ,m\right)}=\frac{1}{K-m+1}\overset{K-m+1}{\underset{i=1}{\sum }}C_{i}^{\left(\tau ,m\right)}\left(\gamma \right). \end{array}$$

Similarly, *C*^(τ, *m*+1)^ is obtained for the sequence ${\left\{w_{j}^{\left(\tau \right)}\right\}}_{j=1}^{K}$ that embedding *m* + 1 adjacent points into the sequence ${\left\{w_{k}^{\left(\tau \right)},w_{k+1}^{\left(\tau \right)},\ldots ,w_{k+m+1}^{\left(\tau \right)}\right\}}_{k=1}^{K-m}$.

Finally, the MSEn of envelope spectrum, MSESEn, is defined to be the set of sample entropy (SampEn) for the scale τ

(23) $$\begin{array}{l} \begin{array}{c} \text{MSESEn}=\text{SampEn}\left(\tau ,m,\gamma \right)=-\ln\;\frac{C^{\left(\tau ,m+1\right)}}{C^{\left(\tau ,m\right)}}. \end{array} \end{array}$$

### 2.4. Flow of Signal Processing

After the OCEEMD is used to adaptively decompose and reconstruct an sEMG signal, MSESEn is applied to evaluate the state of muscle fatigue. In the OCEEMD, finding an optimal solution of the standard deviation of Gaussian white noise helps the sEMG signal to be decomposed quickly in an appropriate way. Nevertheless, not all sub-signals can be decomposed thoroughly. Thus, we calculate coupling degrees between IMFs to detect whether there is mode mixing between IMFs. When mode mixing is detected, we adjust the residual and then perform the decomposition process again.

Note that the decomposition principle of the CEEMD allows us to use the correlation between an IMF component and a residual at the same level to replace the correlation between adjacent IMF components. This is due to the fact that IMF components is determined by the residual at the previous lever (Lu et al., 2019). This treatment features small number of iteration and high computational efficiency.

**Figure 2:**
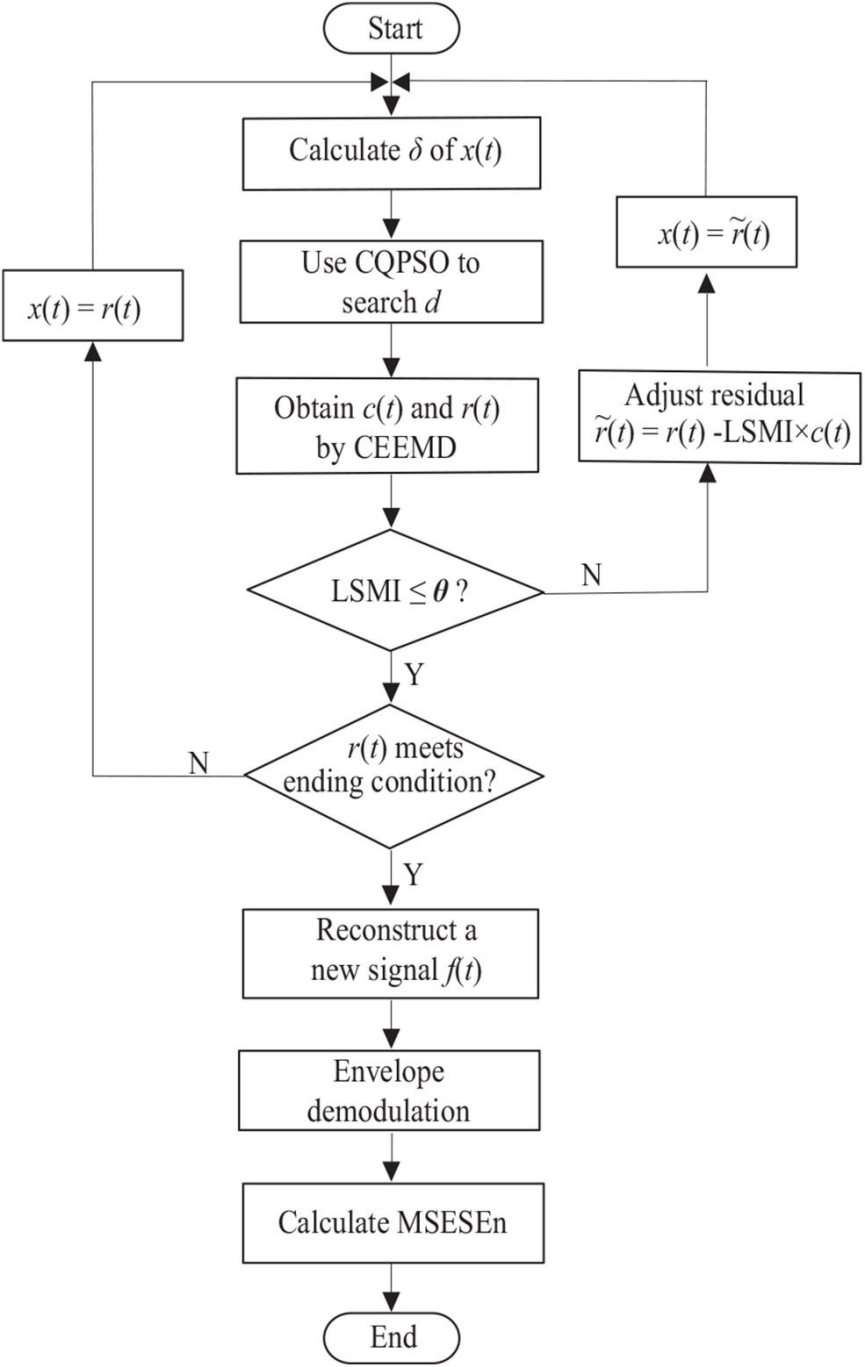
Flow chart of sEMG signal processing.

Summarizing the above explanation gives the following sEMG signal processing steps based on the OCEEMD and MSESEn (Figure 2).

Step 1: Calculate the standard deviation σ of an sEMG signal *x*(*t*). Use the CQPSO to search for an optimal *d* to determine the amplitude of added white noise.
Step 2: Decompose the signal with white noise using the CEEMD and obtain an IMF candidate *c*(*t*) and a residual *r*(*t*).
Step 3: Calculate LSMI between *c*(*t*) and *r*(*t*). Compare LSMI with the selected threshold θ empirically. If LSMI ≤ θ, keep this *c*(*t*) as an effective IMF and go to Step 4. Otherwise, discard this *c*(*t*) as an invalid IMF and go to Step 5 to adjust the residual *r*(*t*).
Step 4: Check whether or not *r*(*t*) meets the terminal condition, that is, whether or not *r*(*t*) is a monotonous function (Yeh et al., 2010). If it is, go to Step 6. Otherwise, take this *r*(*t*) as a new signal *x*(*t*) and go to Step 1 to start another level of decomposition.
Step 5: Remove the overlapping residual information using
  (24) $$\begin{array}{l} \tilde{r}\left(t\right)=r\left(t\right)-\text{LSMI}\times c\left(t\right), \end{array}$$
  take this new residual $\tilde{r}\left(t\right)$ as *x*(*t*), and go to Step 1.
Step 6: Calculate the spectrum of each IMF component, reconstruct a new sEMG signal, *f*(*t*).
Step 7: Obtain the envelope of the reconstructed signal, *f*(*t*), by calculating the demodulation spectrum based on the HHT.
Step 8: Calculate the MSESEn, and thus extract the feature.

In Step 3, if there is LSMI ≤ θ, which means there is little overlapping between *c*(*t*) and *r*(*t*), *c*(*t*) and *r*(*t*) are orthogonal. On the other hand, if there is LSMI > θ, it means that modes are mixing between *c*(*t*) and *r*(*t*).

## 3. Results

The presented method was used to process sEMG signals of all the ten subjects. The processing results of a sEMG signal sampled from the rectus femoris of one subject (age: 39, height: 163 cm, weight: 60 kg) is used as an example to show the typical results.

### 3.1. Acquired Signal

**Figure 3:**
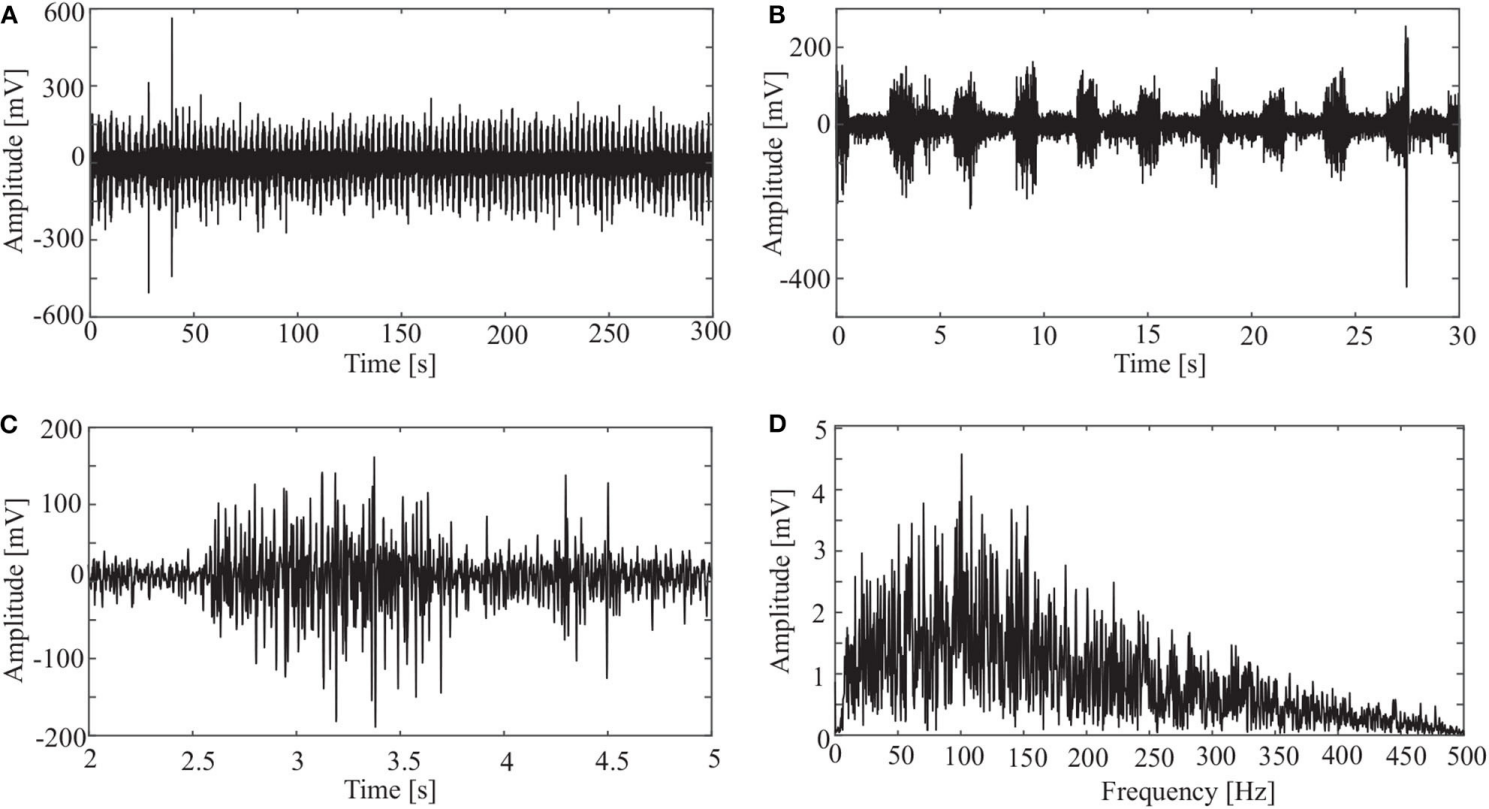
Original sEMG signal and processing result: **(A)** sEMG for the whole length, **(B)** sEMG for the first 30 s, **(C)** sEMG from 2 to 5 s, and **(D)** spectrum of sEMG from 2 to 5 s.

sEMG signals were sampled at a frequency of 1000 Hz. An original signal is shown in Figure 3. The horizontal axis is the sampling time, and the vertical axis is the amplitude of the sEMG. Figure 3A shows the sEMG signal for the whole length (*N* = 300, 000). Figure 3B shows the signal for the first 30 s. It is clear from the figure that the period of the wave is about 3 s. Considering that a period of wave reveals the pedal movement, we chose the signal from 2 to 299 s for processing. Thus, the first period is from 2 to 5 s (Figure 3C), and there are 99 periods for the whole data set.

It is clear from Figure 3D that most frequency components of the sEMG signal are in the range [10, 200] Hz.

### 3.2. Signal Decomposition and Reconstruction

**Figure 4:**
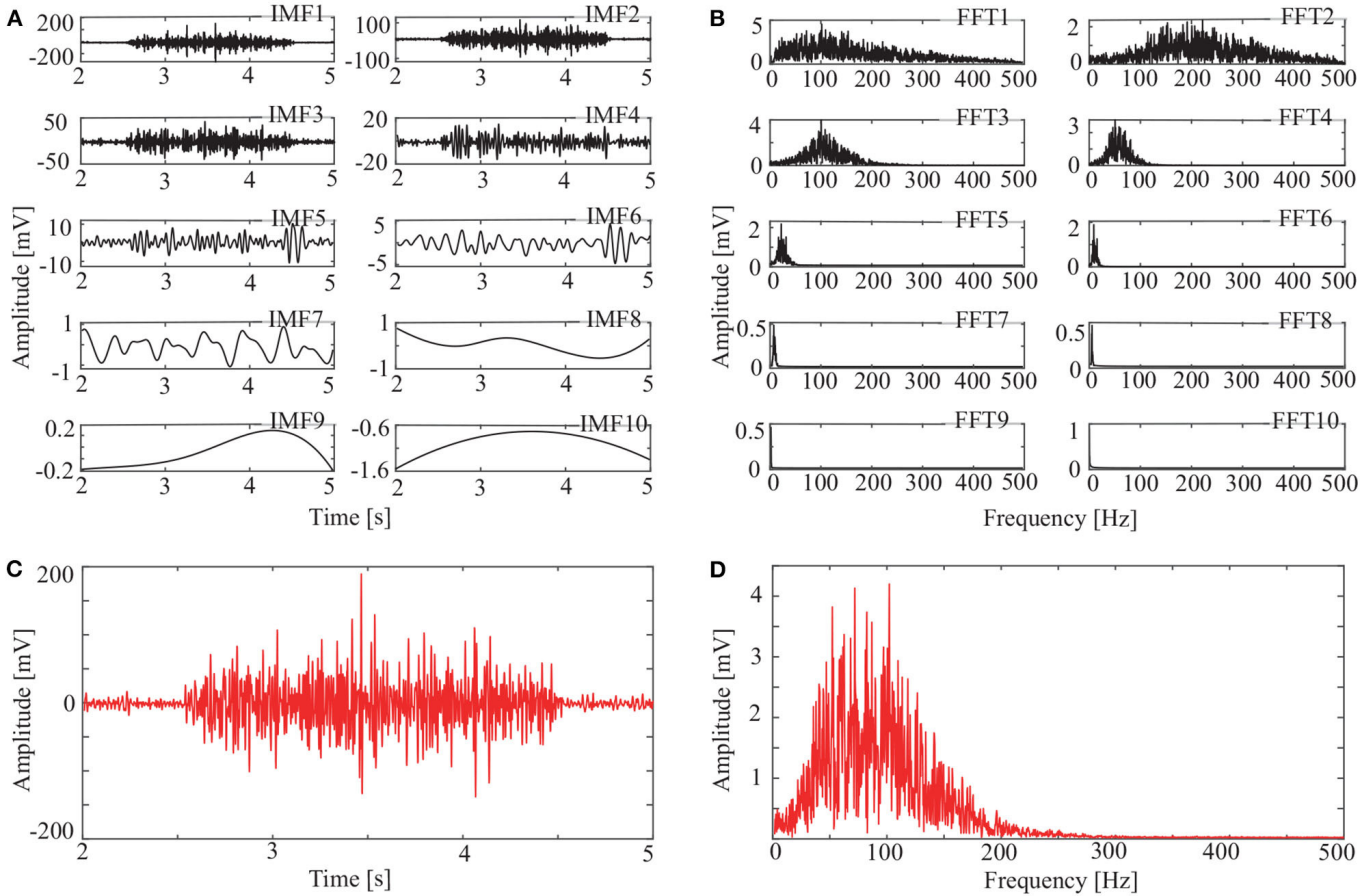
IMFs and spectrums using CEEMD: **(A)** IMFs of signal in Figure 3C, **(B)** Corresponding FFTs, **(C)** Reconstructed signal, and **(D)** Spectrum of reconstructed signal.

**Figure 5:**
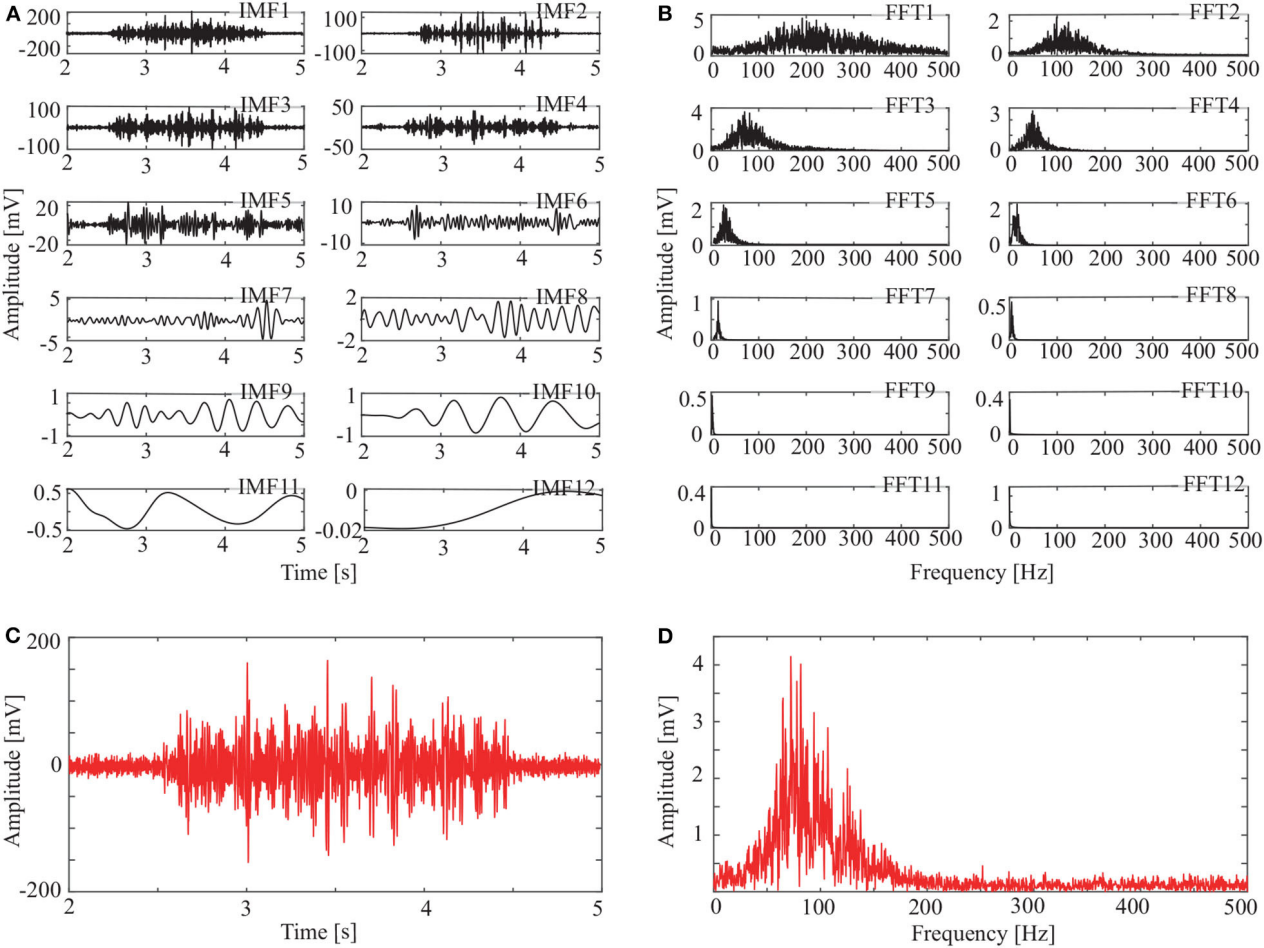
IMFs and spectrums using CEEMDAN: **(A)** IMFs of signal in Figure 3C, **(B)** corresponding FFTs, **(C)** reconstructed signal, and **(D)** spectrum of reconstructed signal.

**Figure 6:**
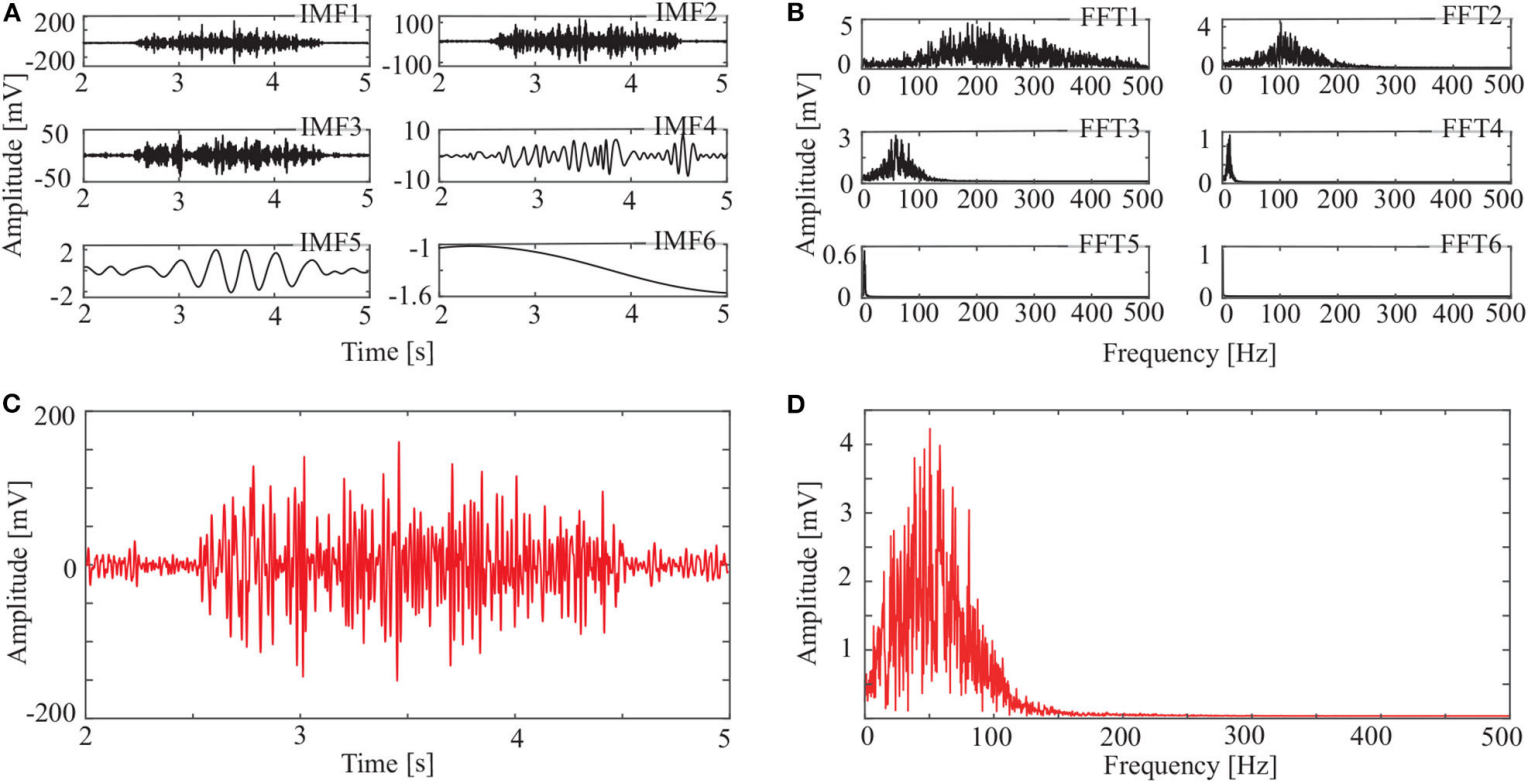
IMFs and spectrums using OCEEMD: **(A)** IMFs of signal in Figure 3C, **(B)** corresponding FFTs, **(C)** reconstructed signal, and **(D)** spectrum of reconstructed signal.

In addition to the OCEEMD, we also used the CEEMD and the CEEMDAN for comparison. Decomposing the signal in Figure 3D and obtained a series of IMFs (Figures 4A, 5A, 6A). These IMFs represent the characteristics of the original signal on different scales. Next, we got the spectrum of each IMF by fast Fourier transform (FFT) (Figures 4B, 5B, 6B). Then, we reconstructed a signal using effective IMFs extracted based on useful spectral distributions (Figures 4C, 5C, 6C), and obtained the corresponding spectrums (Figures 4D, 5D, 6D).

The spectrum of the second IMF in Figure 4B and that of the second and third IMFs in Figure 5B are mixed with other modes. But this does not appear in Figure 6B. This shows that mode mixing is suppressed more successfully for the OCEEMD than that for the CEEMD and the CEEMDAN.

sEMG signals are concentrated within a certain frequency. The highest frequency is about 200 Hz in Figure 4D, 180 Hz in Figure 5D, and 150 Hz in Figure 6D. It suggests that the reconstructed signal in Figure 6C has the minimum noise, which shows that the OCEEMD outperforms the CEEMD and the CEEMDAN.

The qualitative and quantitative analysis was carried out to compare the three methods using the root mean square error (RMSE), the number of IMF components, and the standard deviation of the amplitude ratio, *d* (Table 2). The results reveal the follows:

(1) RMSE of the reconstructed signal is 2.8986 × 10^−4^ for the OCEEMD. It is much smaller than those for the CEEMD and the CEEMDAN. Note that the smaller RMSE is, the difference between the de-noised sEMG signal and the original signal is. So, it means that the reconstructed signal using the OCEEMD has the highest accuracy.
(2) The number of IMFs is 6 for the OCEEMD. It is much smaller than those for the other two methods. This suggests that the computational efficiency of the OCEEMD is higher than that of the CEEMD and the CEEMDAN.
(3) *d* was adjusted in the OCCEMD and the CEEMDAN to adaptively process decomposition, while it was fixed in the CEEMD. A comparison between the CEEMD and the OCEEMD shows that only the first value of *d* is larger for the OCEEMD than for the CEEMD, and the others are all smaller for the OCEEMD than for the CEEMD. A comparison between the CEEMDAN and the OCEEMD shows that only the first value of *d* is the same for both methods, and the others are all smaller for the OCEEMD than for the CEEMDAN. It shows that the OCEEMD has stronger adaptability than the other two methods.
(4) The LSMIs of adjacent IMFs are less than 0.1 for the OCEEMD. They are much smaller than those for the other two. The adjacent IMFs of the OCEEMD have the minimum coupling degrees, indicating that the OCEEMD suppresses the mode mixing very well.

**Table 2:** Comparison of CEEMD, CEEMDAN, and OCEEMD.

	**CEEMD**	**CEEMDAN**	**OCEEMD**
RMSE	4.5347 × 10^−2^	1.3275 × 10^−3^	2.8986 × 10^−4^
No. of IMFs	10	12	6
*d*	±0.27	0.32 0.37 0.28 0.22 0.21 0.16 0.11 0.11 0.09 0.07 0.03	±0.32 ±0.26 ±0.18 ±0.12 ±0.12
LSMI of adjacent IMFs	0.5751 0.5826 0.3529 0.2529 0.1721 0.0819 0.0525 0.0427	0.7238 0.6147 0.4629 0.4588 0.3025 0.3126 0.1196 0.1064 0.1012 0.0828	0.0911 0.0866 0.0562 0.0104

In summary, Table 2 reveals that the OCEEMD is superior to the CEEMD and the CEEMDAN while de-noising sEMG signals.

### 3.3. Extracted Entropy

**Figure 7:**
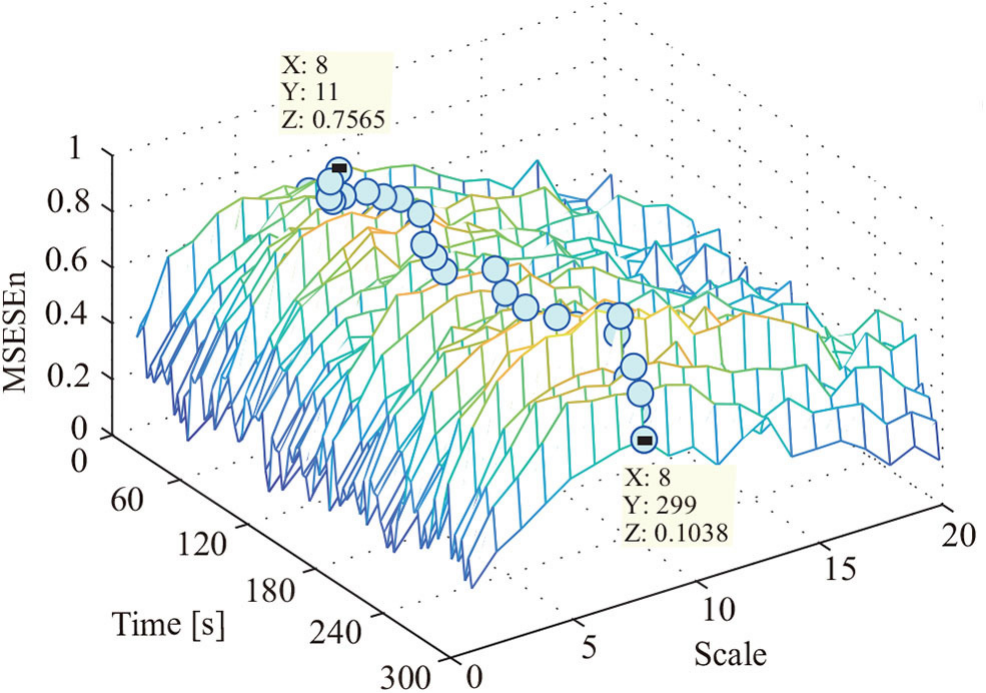
MSESEn of reconstructed signal using OCEEMD with different scales.

This study used the MSEEn to quantify the envelope spectrum of a signal containing 99 periods in the whole pedaling process. We took a coarse-graining processing to each data sequence with different τ (τ ∈[1, 20]) (Figure 7).

**Figure 8:**
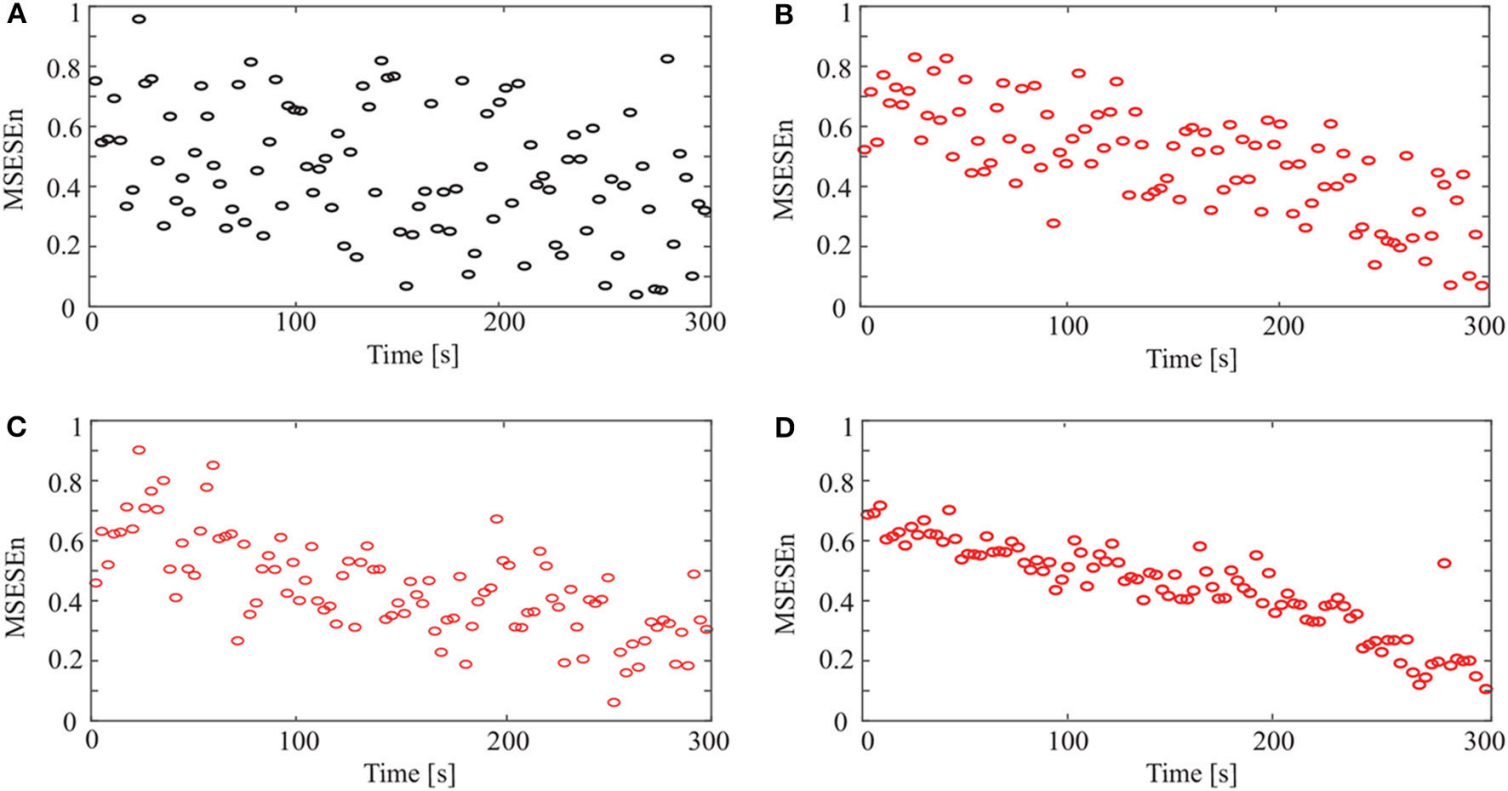
MSESEn of original and reconstructed signals for different methods: **(A)** original signal, **(B)** CEEMD, **(C)** CEEMDAN, and **(D)** OCEEMD.

In Figure 7, the relationships between the MSESEn, the time, and the scale τ show that the MSESEn is low for τ in the range [1, 3] and [15, 20]. The MSESEn is the largest when τ = 8. It means that this scale has the largest correlation with the sEMG signals. Picking out the relationship between the MSESEn and the time for τ = 8 gives Figure 8D. It shows that the MSESEn decreases from 0.7565 to 0.1038 along time. This shows a clear declining trend.

Then we calculated the MSESEn of the original signal for τ = 8 in Figure 3A. The distribution of the MSESEn (Figure 8A) is scattered and is hard to judge the tendency. The MSESEn of the original signal does not show a distinctive feature. However, if we calculated the MSESEn of the reconstructed signals in Figures 4C, 5C, 6C, we can easily observe the correlation of the data and the trend of the muscle fatigue from the distributions (Figures 8B–D). Moreover, we can observe that the regularity and the decreasing tendency of the MSESEn in Figure 8D is more obvious than that in Figures 8B,C. MSESEn clearly shows the changing process of the muscle from fresh to fatigue. It has the advantages of high concentricity, good monotonicity, and relative consistency. These results also show that OCEEMD is an effective preprocessing method for extracting the MSESEn.

**Figure 9:**
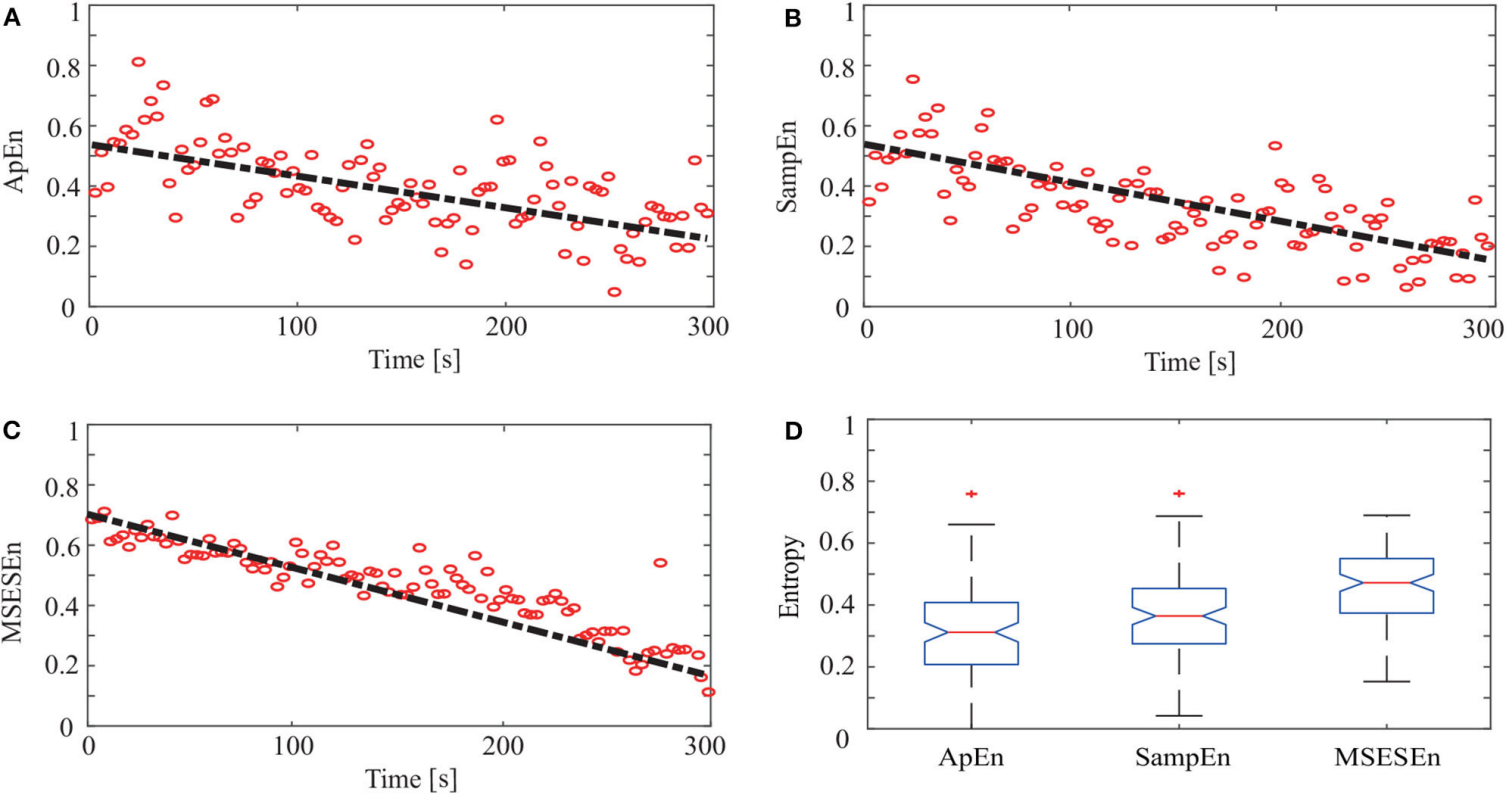
ApEn, SampEn, and MSESEn of OCEEMD: **(A)** ApEn (gradient: −0.0034), **(B)** SampEn (gradient: −0.0038), **(C)** MSESEn (gradient: −0.0051), and **(D)** box diagram.

Then, we quantitatively analyzed two other indicators, ApEn and SampEn, and compared them with the MSESEn for the reconstructed sEMG signal processed by the OCEEMD. Figures 9A–C show the distributions and the best fitting straight lines for the three indicators. A comparison of them shows that MSESEn in Figure 9C declines more significantly and its distribution is more concentrated than ApEn in Figure 9A and SampEn in Figure 9B during the development of muscle fatigue.

Table 3 lists the gradients and intercepts of the trend lines in Figures 9A–C. The absolute gradient of MSESEn is 0.0051. It is 1.5 times larger than that of ApEn and more than 1.3 times larger than that of SampEn. The intercept of MSESEn is 0.57. It is larger than those of the ApEn and SampEn. These values figure that, among these three indicators, the MSESEn dropped the most, suggesting the MSESEn are the most sensitive to the generation of muscle fatigue. Furthermore, Figure 9D shows a box diagram of the three indicators. Clearly, the MSESEn has the least data dispersion and thus the best robustness among the three indicators.

**Table 3:** Gradients and intercepts for different indicators in Figure 8.

	**Gradient**	**Intercept**
ApEn	−0.0034	0.32
SampEn	−0.0038	0.39
MSESEn	−0.0051	0.57

### 3.4. Statistical Analysis

The goodness of fit, *R*^2^, was used to test 40 sEMG signals (four muscles for each of the ten subjects). It measured whether our method effectively extracted the features of the sEMG signals. It is defined to be

(25) $$\begin{array}{l} \begin{array}{c} R^{2}=\frac{\overset{n}{\underset{i=1}{\sum }}y_{i}^{2}-\overset{n}{\underset{i=1}{\sum }}{\left(y_{i}-{ŷ}_{i}\right)}^{2}}{\overset{n}{\underset{i=1}{\sum }}y_{i}^{2}}, \end{array} \end{array}$$

where *y*_*i*_ is the *i*th extracted entropy and ŷ_*i*_ is the corresponding value on the fitting line. We calculated *R*^2^ with the three preprocessing methods and three feature extraction methods. The average of *R*^2^, $\bar{R^{2}}$, with different methods is shown in Table 4.

**Table 4:** $\bar{R^{2}}$ with different methods.

	**CEEMD**	**CEEMDAN**	**OCEEMD**
ApEn	0.8408	0.8613	0.8996
SampEn	0.8914	0.9039	0.9372
MSESEn	0.9423	0.9771	0.9806

It is clear from Table 4 that, compared with the CEEMD and the CEEMDAN, the OCEEMD yields the largest $\bar{R^{2}}$ for three different definitions of entropy. This indicates that the OCEEMD extracts the entropy more effectively than the other two methods do. Moreover, the MSESEn, which is the new one used in this study, has the largest $\bar{R^{2}}$ than the ApEn and the SampEn. This shows the advantage of using this entropy in this study.

## 4. Discussion

The non-linear methods were explored to process the sEMG signals. Figures 4, 5, 6, and Table 2 show that the CEEMD, the CEEMDAN, and the OCEEMD have their merits. While the number of IMFs is smaller for the CEEMD than for the CEEMDAN, the de-noising effect is better for the CEEMDAN than for the CEEMD. The features closely relate to their decomposition processes as well as the intermittent and spectral characteristics of sEMG signals. The CEEMD adds *N* sets of Gauss white noise with the same amplitudes but opposite signs to an original sEMG signal, and separately decomposes the 2*N* new signals into mode components (Yeh et al., 2010). Since there may have asymmetric mode components in sEMG signals, some noise may remain in the signals when summing up and averaging the components. The CEEMDAN adaptively adds white noise to an original signal and yields the IMFs by obtaining a unique residual at each decomposition level (Torres et al., 2011). This method ensures that the noise does not transfer from the present decomposition level to the next level, but the computational expense is high and this method has some dummy IMFs.

Then, the OCEEMD further improves the performance. For example, the number of IMFs for the OCEEMD is 38% less than that for the CEEMD. This indicates the OCEEMD has high decomposition efficiency. It might be due to that the OCEEMD embedded the CQPSO and thus quickly found the optimal solution of *d*. However, the *d* at the first level is larger. The reason is that the standard deviation of the reconstructed sEMG signal after using the OCEEMD is very small, which indicates the reconstructed sEMG signal after using the OCEEMD was de-noised very well. Other values of *d* are smaller, validating the adaptability of the OCEEMD. For the LSMI, the correlations between each IMFs are reduced by more than 80%. The results are inseparable from the threshold criterion of the LSMI on each level. The crucial step was only IMFs with very small LSMI could be further decomposed to the next level. So, every adjacent IMFs were irrelevant finally. Thus, there was no mode mixing in the whole decomposition.

Figure 8 and Table 4 show that the OCEEMD is superior to the CEEMD and the CEEMDAN. The MSESEn reveals the trend of muscle fatigue. The trend can hardly be observed from the MSESEn in Figure 8A because the original signal was not preprocessed. Using the CEEMD, the CEEMDAN, and the OCEEMD to preprocess the signal explicitly displays the trend. As shown in Figures 8B–D, the MSESEn in Figures 8B–D decreases over time. This indicates that these methods are suitable for processing non-stationary sEMG signals. In particular, the MSESEn for the OCEEMD is well-lumped to show the trend (Figure 8D and Table 4). This is because that the OCEEMD greatly reduces the mode mixing and residual noise of IMFs.

The results in Figures 8B–D shows high consistency with the conclusions in (Pincus, 2006; Pethick et al., 2019). The MSESEn, like ApEn and SampEn, reflects the complexity and the power distribution of sEMG signals in a frequency range (Pincus, 2006; Pethick et al., 2019). When a muscle begins contraction during physical activity, the muscle fibers are activated and show disordered discharge, producing a signal with widely distributed power. At this point, the components of the sEMG signal are complex, and the ratio of the power to total power is large. Thus, the MSESEn is large. After a period of muscle contraction, some muscle fibers are “tired” and the main muscle fibers still participate in the activity. So, the power decreases. It is clear and simple to detect the main components of the signal. Thereby, the MSESEn becomes small. Thus, the decrease of the MSESEn reveals the degree of muscle fatigue.

Compared to the ApEn and the SampEn, the MSESEn has better centrality and robustness. Table 3 shows that the gradient of the MSESEn increased by 33% compared with the ApEn and 25% compared with the SampEn. Tables 3, 4 also show that the MSESEn has the best fitting effect. There are two reasons. First, the OCEEMD decomposes the sEMG signals according to their envelope spectrum characteristics. The MSESEn, which takes into consideration “envelope spectrum entropy,” is sensitive to the changing trend in sEMG signals. Second, sEMG signals have unknown potential sequence patterns and related time scales. The MSESEn, which uses multiple scales, provides additional spatial statistics. So, the MSESEn decreases the most, suggesting that it has the greatest correlation with muscle fatigue levels, and thus it best reflects the changes of muscle fatigue. Furthermore, this obvious downward trend indicates that it has good anti-interference ability and facilitates the feature classification of muscle fatigue in the next step. Therefore, MSESEn is a feasible feature for analyzing muscle fatigue based on sEMG.

Overall, the OCEEMD, which integrates the LSMI and CQPSO, is used to decompose sEMG signals to obtain some IMFs with no mode mixing. This algorithm, along with the next signal reconstruction by useful IMFs, aims to de-noise and purify the signals. Then, the MSESEn of the reconstructed sEMG is calculated to detect the process of muscle fatigue. The test on the decomposition and reconstruction demonstrates that the OCEEMD effectively suppressed mode mixing between IMFs with fast iteration. Experiments on the extracted envelope illustrate that the MSESEn displays muscle fatigue clearly. These results show that our method has the potential to process sEMG signals and measure muscle fatigue.

In this study, we devised the OCEEMD and MSESEn to capture the features of sEMG signals reflecting the changing trend of muscle fatigue. A 5-min pedaling experiment was designed to record the sEMG signals and to verify the effectiveness of our method. On the other hand, experiments for different conditions, such as time duration and pedaling load, may provide us a more comprehensive understanding of the relationship between sEMG signals and muscle fatigue. They will be carried out in the future.

## Data Availability Statement

The raw data were recorded at the Advanced Mechatronics Laboratory, School of Engineering, Tokyo University of Technology, Japan. The data supporting the findings of this study are available from zhaojuan0859@cug.edu.cn or she@stf.teu.ac.jp on request.

## Ethics Statement

The studies involving human participants were reviewed and approved by The ethical committee of Tokyo University of Technology. The patients/participants provided their written informed consent to participate in this study.

## Author Contributions

JZ and JS conceived this study. JZ and KP performed the experiments and wrote the manuscript. JS, EF, DW, and MW contributed to the methodology. JZ, JS, and KP revised the manuscript. All the co-authors agreed with the present version.

## Conflict of Interest

The authors declare that the research was conducted in the absence of any commercial or financial relationships that could be construed as a potential conflict of interest.
